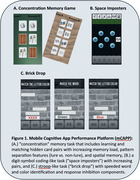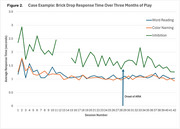# Monitoring Cognition in Patients Undergoing Anti‐Amyloid Therapies with A Remote App‐Based Digital Cognitive Assessment Tool

**DOI:** 10.1002/alz70857_107550

**Published:** 2025-12-24

**Authors:** Dawn Mechanic‐Hamilton, Valerie Humphreys, Danielle Hing, Laura Schankel, Christopher A Brown, David A. Wolk

**Affiliations:** ^1^ Penn Alzheimer's Disease Research Center, University of Pennsylvania, Philadelphia, PA, USA; ^2^ University of Pennsylvania, Philadelphia, PA, USA

## Abstract

**Background:**

Mobile, valid and engaging cognitive assessments are essential for detecting and tracking change in cognition. With the approval of anti‐amyloid therapies (AATs), there is a need to monitor for both benefits and risks of these therapies. This pilot study aims to determine the feasibility and usability of an at‐home, app‐based cognitive assessment, the mobile cognitive app performance platform (mCAPP), to remotely collect cognitive performance data in a cohort of patients with Mild Cognitive Impairment (MCI) or mild dementia due to Alzheimer's disease (AD) undergoing AAT.

**Method:**

mCAPP includes three gamified tasks (Figure 1). Eleven participants with MCI or mild dementia due to AD used mCAPP in the clinic and at home for up to three months while undergoing AAT treatment. They also completed paper and pencil neuropsychological tests and questionnaires about their use of technology and mCAPP usability. Participants included 7 women and 4 men with 15.1±2.6 years of education who were amyloid positive according to either Amyloid PET or cerebrospinal fluid results and deemed eligible for treatment by a multi‐disciplinary AAT consensus panel at the University of Pennsylvania.

**Result:**

Participants played the mCAPP tasks in‐clinic and consistently engaged in 1‐3 sessions per week for up to 3 months at‐home. All participants used a smartphone in their daily lives. mCAPP usability rating was 7.1±1.2 (0‐9 scale). Most participants rated the difficulty of the tasks as “just right” for Concentration (89%), Brick Drop (78%), and Space Imposters (78%) after playing the games one time. All tasks showed lower performance when comparing low and high cognitive load blocks (*p*'s < .001). mCAPP measures correlated with some, but not all, paper and pencil measures from analogous domains. An example of one patient's performance during AAT treatment is shown in Figure 2.

**Conclusion:**

This pilot study shows mCAPP's usability for both in‐clinic and at‐home use in a cohort of patients undergoing AAT. Performance across measures indicate initial reliability and validity of mCAPP. Future analyses will include the evaluation of mCAPP performance across multiple timepoints, practice effects compared to participants with unimpaired cognition, and longitudinal data analysis over the course of anti‐amyloid treatment.